# Lifetime estrogen exposure and domain-specific cognitive performance: results from the IGNITE study

**DOI:** 10.3389/fnagi.2025.1524474

**Published:** 2025-04-03

**Authors:** Amber Watts, Shannon Donofry, Hayley Ripperger, Nicole M. Eklund, Lu Wan, Chaeryon Kang, George Grove, Lauren E. Oberlin, Swathi Gujral, Eric D. Vidoni, Jeffrey M. Burns, Edward McAuley, Charles H. Hillman, Arthur F. Kramer, M. Ilyas Kamboh, Kirk I. Erickson

**Affiliations:** ^1^University of Kansas Alzheimer’s Disease Research Center, University of Kansas Medical Center, Fairway, KS, United States; ^2^Behavioral Research in Aging Neuroscience Cognition and Health (BRANCH) Lab, Psychology, University of Kansas, Lawrence, KS, United States; ^3^Behavioral and Policy Sciences, Rand Corporation, Pittsburgh, PA, United States; ^4^Brain Aging and Cognitive Health Lab, Psychology, University of Pittsburgh, Pittsburgh, PA, United States; ^5^Behavioral Neuroscience, Department of Graduate Medical Sciences, Boston University School of Medicine, Boston, MA, United States; ^6^Neuroscience, Advent Health Research Institute, Orlando, FL, United States; ^7^Psychiatry, University of Pittsburgh School of Medicine, Pittsburgh, PA, United States; ^8^Psychiatry, Weill Cornell Medicine, New York, NY, United States; ^9^Health and Kinesiology, University of Illinois Urbana-Champaign, Urbana, IL, United States; ^10^The Beckman Institute for Advanced Science and Technology, University of Illinois Urbana-Champaign, Urbana, IL, United States; ^11^Center for Cognitive and Brain Health, Psychology, Northeastern University, Boston, MA, United States; ^12^Human Genetics, School of Public Health, University of Pittsburgh, Pittsburgh, PA, United States

**Keywords:** cognition, aging, women, estrogen, oophorectomy, menopause

## Abstract

**Introduction:**

Disruptions in estrogen exposure (i.e., surgically induced menopause) have been linked to poorer cognitive aging and dementia risk. Hormone therapy use (e.g., birth control, menopausal hormone therapy) has shown mixed associations with cognitive performance, possibly due to limited cognitive test batteries. To address previous inconsistencies, we investigated baseline data from Investigating Gains in Neurocognition in an Intervention Trial of Exercise (IGNITE). We hypothesized that (1) oophorectomy prior to natural menopause would be associated with poorer cognitive performance, (2) timing and duration of birth control and menopausal hormone therapy would influence associations with cognitive performance, and (3) *APOE4* carrier status would interact with oophorectomy and hormone therapy to influence cognitive performance.

**Methods:**

In 461 post-menopausal females (M age = 69.6) we assessed oophorectomy and hormone therapy use to examine associations with the Montreal Cognitive Assessment (MoCA) and factor-analytically derived composite scores for episodic memory, processing speed, working memory, executive function/attentional control, and visuospatial processing.

**Results:**

Hypothesis (1) We did not observe associations between oophorectomy prior to natural menopause and poorer cognitive performance. However, hormone therapy use, started on average within 2 years of oophorectomy, was associated with better episodic memory (β = 0.106, *p* = 0.02), working memory (β = 0.120, *p* = 0.005), and visuospatial processing (β = 0.095, *p* = 0.03). Hypothesis (2) Birth control use was associated with better performance on the MoCA (β = 0.093, *p* = 0.04), working memory (β = 0.102, *p* = 0.02), and executive function/attentional control (β = 0.103, *p* = 0.02). However, duration and timing of birth control and menopausal hormone therapy were not associated with cognitive performance. Hypothesis (3) We did not observe significant interactions between *APOE4* status and oophorectomy or hormone therapy in their associations with cognitive performance.

**Discussion:**

Our results suggest exposure to estrogen during adulthood, specifically birth control and hormone therapy among women undergoing pre-menopausal oophorectomy, benefits cognitive function in older adulthood. Our comprehensive cognitive battery allowed us to examine cognitive function with a high degree of granularity. Future work should evaluate causal mechanisms of associations between lifetime estrogen exposure and later life cognitive function.

## 1 Introduction

Greater lifetime exposure to estrogen, a neuroprotectant, is associated with a reduced risk of age-related cognitive decline and dementia (Arevalo et al., 2015; Ryan et al., 2009). In particular, 17β-estradiol is involved in neural plasticity, adult neurogenesis, and signaling with other neuroprotective factors such as brain derived neurotrophic factor and insulin-like growth factor-1 (Arevalo et al., 2015). Assessment of brain estrogen in living humans is very rare, as it is costly and complex (Mosconi et al., 2024). A more readily measurable indicator of lifetime estrogen exposure is reported use of medications that contain hormones, particularly estradiol, including menopausal hormone therapies. Research indicates that lifetime estrogen exposure is also reflected in characteristics of reproductive and menopausal history. Several of these characteristics, including pregnancy history and reproductive surgeries have been associated with cognitive function across the lifespan, including in older adulthood (Karim et al., 2016; Rocca et al., 2021).

Drawing conclusions from the literature about associations between hormone therapy and cognitive performance is challenging for several reasons including the complexity of differing types of therapies (e.g., estrogen only, estrogen plus progesterone) and timing of hormone therapy. A recent systematic review and meta-analysis examining the effects of menopause hormone therapy on cognition in randomized controlled trials concluded that overall there were no effects on cognitive domain scores (Andy et al., 2024). However, three caveats were emphasized. First, estrogen therapy improved global cognition after surgical menopause (bilateral oophorectomy, i.e., surgical removal of both ovaries) compared to placebo. Second, the timing of estrogen therapy initiation matters. Specifically, estrogen initiated near menopause onset was associated with improved verbal memory, while later initiation had no effects. Longer durations of estrogen use were associated with worsening in some cognitive domains including visual memory. This reinforces the “critical window” hypothesis that optimal timing of estrogen therapy exposure is around the time of menopause when estrogen levels are changing, but before other age-related brain changes occur. Finally, the combination of estrogen plus progesterone had positive effects when taken in midlife, near to the age of menopause, but negative effects when taken in later life, often defined as after age 60. Long term use of hormonal contraceptives have also been associated with better global cognitive performance and verbal memory (Karim et al., 2016).

Surgically induced menopause, specifically bilateral oophorectomy, has been repeatedly associated with poorer cognitive function, especially when it occurs early relative to the typical age of menopause onset (Rocca et al., 2021). The suspected rationale for this is the sudden dramatic reduction of estrogen, particularly prior to the natural onset of menopause. This explanation is supported by studies showing that the degree of cognitive risk or decreased function is associated with age at surgical intervention, and subsequent use of estrogen therapy (Bove et al., 2014). The timing and duration of estrogen therapy appear to influence the cognitive benefits (Bove et al., 2014; Hogervorst and Bandelow, 2007). Though less well-established, some studies suggest unilateral oophorectomy and hysterectomy with conservation of ovaries are associated with lower lifetime estrogen exposure, and thus may also confer cognitive risks (Crawford, 2022; Phung et al., 2010).

The apolipoprotein E (*APOE*) 4 allele is the strongest known genetic risk factor for Alzheimer’s disease and evidence suggests the risk is greater in women than in men (Neu et al., 2017). This vulnerability is dependent on interactions between estrogens and *APOE* genotype (Valencia-Olvera et al., 2023). Specifically, *APOE* modulates systemic and neural outcomes of menopause and estrogen-based hormone therapy. A recent study using UK Biobank data reported that younger age at oophorectomy, particularly in *APOE4* carriers, conferred higher odds of developing Alzheimer’s disease over a longitudinal follow up (Calvo et al., 2024).

Results of studies of hormone therapy and cognition as it relates to *APOE* genotype have been mixed, for example, suggesting hormone therapy improved cognitive function and brain volume for *APOE*4 carriers (Saleh et al., 2023), or conversely that hormone therapy benefitted cognition only in non-*APOE4* carriers (Yaffe et al., 2000). One possible explanation for these contradictory findings is that timing of estrogen exposure matters for understanding effects on cognitive function and potential interactions with *APOE* genotype. In the study reporting greater benefits of estrogen therapy for *APOE4* carriers (ages 50+), younger age of estrogen therapy initiation was associated with greater brain volumes cross-sectionally (Saleh et al., 2023). Whereas, in the study reporting cognitive benefits in non-*APOE4* carriers, current hormone use in postmenopausal women (ages 65+) was associated with less longitudinal decline on a dementia screening tool (Yaffe et al., 2000).

Given these complexities and the inconsistent findings documented in prior literature, organizations such as The Menopause Society and the Women’s Alzheimer’s Movement have called for additional research to elucidate menopause and hormone-related mechanisms for cognitive decline and dementia. There is also broad recognition that samples with greater demographic (e.g., racial) heterogeneity are needed to maximize the generalizability of findings. Thus, the present study aimed to address some of these mixed findings in a large and racially diverse sample of cognitively well-characterized women, that includes measures of menopausal history and *APOE* genotype. Specifically, we evaluated the following hypotheses: (1) surgically induced menopause (i.e., oophorectomy) will be associated with poorer cognitive performance and depend on timing of surgical intervention, (2) the timing and duration of menopausal hormone therapy and hormone-based birth control will influence associations with cognitive performance, and (3) *APOE4* carriers with a history of pre-menopausal oophorectomy will have poorer cognitive performance, while *APOE4* carriers exposed to hormone therapy will have better cognitive performance.

## 2 Materials and methods

### 2.1 Study design

The IGNITE study (Investigating Gains in Neurocognition in an Intervention Trial of Exercise: NCT02875301, R01AG053952) was a multi-center (Pittsburgh, Boston, Kansas City) randomized clinical trial that aimed to examine whether a 12 months aerobic exercise intervention would improve cognitive performance and neuroimaging markers of brain health in sedentary, cognitively unimpaired older adults in a dose-dependent manner. The present study is a secondary analysis of baseline data from the IGNITE study.

### 2.2 Participants

Recruitment strategies targeted community samples through newspapers, health system research registries, direct mailings, senior centers and churches, and online media, and have been previously described (Vidoni et al., 2021). Participants were enrolled on a rolling basis between 2017 and 2022 with recruitment of racially and ethnically underrepresented participants proportional to the demographic characteristics of each study site. Female participants made up 71.1% of the total study sample. The present study includes data from only female participants (*N* = 461). Detailed study inclusion criteria are published elsewhere (Erickson et al., 2019). Briefly, participants were required to be 65–80 years old, relatively physically inactive (i.e., exercise less than 3 days per week, and exercise less than 20 min per day on days when they do exercise), within a normal range on cognitive assessments within broad limits, and able to safely engage in moderate intensity exercise.

### 2.3 Measures

#### 2.3.1 Reproductive history and hormone therapy

Female participants completed a questionnaire asking about menopause and reproductive history. This included self-reported information about whether participants experienced natural menopause, and whether they had undergone hysterectomy and/or oophorectomy. Participants reported whether they had ever used birth control or hormone therapy, and if they had used hormone therapy, whether it was used in the last 3 months. Participants reported current medications which we classified according to the Anatomical Therapeutic Chemical (ATC) system and the Defined Daily Dose (DDD) as a unit of measure according to the World Health Organization methodology. In the present study, we included medications classified as G3, indicating that they contain “sex hormones and modulators of the genital system” such as estradiol. Some participants who reported using a hormone-based medication did not report the medication type, but these were still included.

#### 2.3.2 Menopausal status

Using three pieces of information (self-reported natural menopause, hysterectomy, oophorectomy), we categorized participants into categories: natural menopause (cessation of menses for 12+ months without removal of ovaries or uterus), oophorectomy (defined in our study as removal of ovaries with or without hysterectomy), hysterectomy without oophorectomy (removal of uterus without removal of ovaries, menstrual bleeding ceased and ability to report continued ovulation absent), and unclear (no report of hysterectomy or oophorectomy, natural menopause is uncertain).

Of note, self-reports are likely based on observation of bleeding as other indicators of menstruation are more difficult to observe. There are other causes of vaginal bleeding besides menstruation (e.g., fibroids). Participants are unlikely to have experienced reproductive surgery without being able to report it, but it may occasionally be unclear what procedures occurred and for what reasons. Thus, we observed several sources of potential error related to self-report in determining menopause type which are described in Appendix 1. Lord et al. (2009) support the use of self-report measures for indicators of lifelong estrogen exposure.

#### 2.3.3 Cognitive assessment

Participants completed a comprehensive cognitive evaluation consisting of the Montreal Cognitive Assessment (MoCA) and measures of processing speed, working memory, episodic memory, executive function/attentional control, and visuospatial processing. A full list of the tests is presented in Table 1. Performance in each domain was measured using previously established latent factors derived from a confirmatory factor analysis (Oberlin et al., 2025), with higher values reflecting better performance. Baseline assessment visits lasted approximately 4 h and were administered by annually certified psychometricians and split across 2 days of testing to prevent fatigue and frustration. The test battery included neuropsychological tests for the purposes of adjudication of cognitive status and for comprehensive assessment of cognitive function. Only cognitively unimpaired participants were included.

**TABLE 1:** Neuropsychological tests in each cognitive factor.

Domain	Test
Episodic memory	Logical memory (Tulsky et al., 2003)*
	Paired associates (Salthouse et al., 1996)*
	MoCA delayed recall (Nasreddine et al., 2005)
	Picture sequence (Zelazo et al., 2013)^
	Hopkins Verbal Learning Test (HVLT) (Brandt, 1991)
	Brief Visuospatial Memory Test – Revised (BVMT) (Benedict et al., 1996)
Processing speed	Digit Symbol Substitution Test (DSST) (Wechsler, 2008)
	Trail Making Test, Part A (Reitan, 1958)
	Letter comparison (Salthouse and Babcock, 1991)*
Working memory	Spatial working memory (Erickson et al., 2011)
	N-back (Drake et al., 2022)
	List sorting working memory (Zelazo et al., 2013)^
Executive function/attentional control	Trail Making Test, Part B (Reitan, 1958)
	Dimensional Change Card Sort (DCCS) (Zelazo et al., 2013)^
	Stroop (Stroop, 1935)
	Flanker (Zelazo et al., 2013)^
Visuospatial	Spatial relations (Bennett et al., 1947)*
	MoCA clock draw (Nasreddine et al., 2005)
	Matrix reasoning (Raven, 1962)*

*The version of the test that was adapted by Salthouse and colleagues as part of the Virginia Cognitive Aging Project.

^From the NIH Toolbox, version 2.

#### 2.3.4 *APOE* genotyping

The common *APOE* polymorphism has three alleles (*APOE2*, *APOE3*, *APOE4*). Carriers of the *APOE4* allele have an increased risk of Alzheimer’s disease, with homozygous (4/4 genotype) having the greatest elevation in risk (Yamazaki et al., 2019). Genotypes for the two *APOE* SNPs resulting in six genotypes was performed on DNA samples using TaqMan assays (Fan et al., 2023). Participants with at least one *APOE4* allele (2/4, 3/4, 4/4) were classified as *APOE4* carriers.

#### 2.3.5 Participant demographics

Age, socioeconomic status (SES), and years of education were determined by participant self-report. A composite score reflecting SES was generated from measures of income, savings, debt-adjusted savings, and financial stability from the MacArthur Socioeconomic Status Index (Seeman et al., 2004). Description of the creation of this composite score can be found in pre-print form at the following link https://papers.ssrn.com/sol3/papers.cfm?abstract_id=5062727. Age, SES, and years of education were included as covariates in our analyses.

#### 2.3.6 Statistical analysis

We conducted descriptive analysis to represent proportions of participants in subgroups (e.g., those with and without oophorectomy). We used chi-squared tests to evaluate whether differences in dichotomous outcomes (i.e., taking a medication) between groups (i.e., oophorectomy) were greater than expected due to chance. We reported the X^2^ value, the degrees of freedom, and the *p*-value. We conducted a series of multiple linear regression models to estimate the relationships between reproductive history variables (i.e., oophorectomy, birth control use, menopausal hormone therapy) and cognitive performance accounting for covariates including age, years of education, *APOE4* carriage, and SES. We estimated change in R^2^ to indicate the proportion of variance in the outcome attributable to significant predictors of interest. We used the Benjamini-Hochberg correction with a false discovery rate (FDR) of 0.05 to correct for inflation of type 1 error associated with multiple testing (Benjamini and Hochberg, 1995). Specifically, for each hypothesis, we estimated corrected *p*-values for the effect of all variables in the model (i.e., predictors and covariates) across six outcome variables (five cognitive domains and the MoCA). We chose Benjamini-Hochberg as our method for correction because it has been shown to preserve statistical power substantially and be less punitive than traditional family wise error correction methods (Benjamini and Hochberg, 1995). To examine possible moderating effects, we included interaction terms in the linear regression analyses (i.e., hormone therapy use x *APOE* carrier status). We examined multicollinear tolerance indices in linear regression models. The hypotheses were tested according to the following multiple linear regression equations:

Hypothesis 1: *Cognitive Test Score* = β *o* + β_1_*age*_1_ + β_2_*education*_2_ + β_3_*SES*_3_ + β_4_*APOE*4_4_ + β_5_*oophorectomy*_5_ + β_6_*hormone therapy use*_6_ + ε

Hypothesis 2a: *Cognitive Test Score* = β*o* + β_1_*age*_1_ + β_2_*education*_2_ + β_3_*SES*_3_ + β_4_*APOE*4_4_ + β_5_*birthcontrol*_5_ + ε

Hypothesis 2b: *Cognitive Test Score* = β*o* + β_1_*age*_1_ + β_2_*education*_2_ + β_3_*SES*_3_ + β_4_*APOE*4_4_ + β_5_*hormone therapy*_5_ + β_6_*duration of hormone therapy*_6_ + β_7_*age started hormone therapy*_7_ + β_8_*menopause age minus hormone therapy start age*_8_ + β_9_*birth control*_9_ + ε

Hypothesis 3a: *Cognitive Test Score* = β*o* + β_1_*age*_1_ + β_2_*education*_2_ + β_3_*SES*_3_ + β_4_*APOE*4_4_ + β_5_*oophorectomy*_5_ + β_6_*oophorectomy* * *APOE*4*interaction*_6_ + ε

Hypothesis 3b: *Cognitive Test Score* = β*o* + β_1_*age*_1_ + β_2_*education*_2_ + β_3_*SES*_3_ + β_4_*APOE*4_4_ + β5*birth control*_5_ + β_6_birth control*APOE4*interaction*_6_ + ε

Hypothesis 3c: *Cognitive Test Score* = β*o* + β_1_*age*_1_ + β_2_*education*_2_ + β_3_*SES*_3_ + β_4_*APOE*4_4_ + β_5_*hormone therapy*_5_ + β_6_hormone therapy*APOE4*interaction*_6_ + ε

## 3 Results

### 3.1 Participant demographic characteristics

The present study included *N* = 461 female participants from the IGNITE study between the ages of 65 and 80 years. Demographic characteristics of the sample are presented in Table 2. Based on self-report data in response to survey items regarding menopause experiences, we categorized female participants into four categories of menopause type (Table 2).

**TABLE 2:** Demographic characteristics.

	M (SD)	*N* (%)
Age (years)	69.58 (3.61)	–
Education (years)	16.24 (2.22)	–
*APOE4*		
Carrier	–	129 (28.4)
Non-carrier	–	326 (71.6)
Missing	–	6 (1.3)
Race		
African American	–	106 (23.0)
Asian	–	8 (1.7)
Caucasian	–	333 (72.2)
Native Hawaiian/ Pacific Islander	–	1 (0.2)
Biracial	–	10 (2.2)
Another race	–	2 (0.4)
No response	–	1 (0.2)
Menopause status		
Spontaneous menopause	–	288 (62.7)
Oophorectomy	–	107 (23.2)
Hysterectomy, no oophorectomy	–	53 (11.5)
Unclear	–	11 (2.4)
Missing	–	2 (< 1)

Cases of “unclear” menopause were included in models as participants without history of oophorectomy according to their reported use of hormone-based medication.

### 3.2 Hypothesis 1 oophorectomy prior to natural menopause

We hypothesized that surgically induced menopause (i.e., oophorectomy) prior to natural menopause would be associated with poorer cognitive performance. In our sample, 107 participants (23.2% of females) reported undergoing oophorectomy, with 73 participants (15.8%) undergoing oophorectomy prior to natural menopause, at an average age of 40.18 (SD = 8.7). Of women who underwent oophorectomy prior to natural menopause, 46 (63.0%) reported using hormone therapy, including vaginal creams, at some point in their lives, with 70.2% of those starting within 2 years of the oophorectomy, and lasting an average duration of 7.8 years (SD = 12.2). The average age of staring hormone therapy for this group was 44.0 (SD = 6.5). Participants who underwent oophorectomy prior to menopause were significantly more likely to report taking a G3 class medication [X^2^ (df) = 9.1 (1), *p* = 0.003]. Adjusting for age, education, SES, *APOE4* carrier status, and hormone therapy use, oophorectomy prior to natural menopause was not significantly associated with cognitive performance in any domain (Table 3). Among this group with pre-menopausal oophorectomy, hormone therapy use was associated with significantly better performance on tests of episodic memory (r^2^ change = 0.010), working memory (r^2^ change = 0.013), and visuospatial processing (r^2^ change = 0.006).

**TABLE 3:** Association of oophorectomy with cognitive performance, adjusting for covariates.

	MoCA		Episodic memory		Processing speed		Working memory		Executive function/ attentional control		Visuospatial processing	
	**β**	***P***	**β**	***P***	**β**	***P***	**β**	***P***	**β**	***P***	**β**	***P***
Age	-0.148	<0.001	-0.159	<0.001	-0.307	<0.001	-0.291	<0.001	-0.325	<0.001	-0.238	<0.001
Education	0.229	<0.001	0.251	<0.001	0.178	<0.001	0.255	<0.001	0.211	<0.001	0.300	<0.001
SES	0.165	<0.001	0.119	0.012	0.216	<0.001	0.193	<0.001	0.190	<0.001	0.164	<0.001
*APOE4* carrier	-0.129	0.004	-0.114	0.010	-0.035	0.416	-0.060	0.151	-0.030	0.474	-0.023	0.587
Oophorectomy	0.048	0.291	0.019	0.670	-0.044	0.316	-0.044	0.303	-0.057	0.192	-0.062	0.155
Hormone therapy	0.077	0.089	0.106	0.020	0.044	0.320	0.120	0.005	0.080	0.067	0.095	0.029
Adjusted R^2^	0.141	–	0.136	–	0.193	–	0.229	–	0.212	–	0.211	–

Corrected for type 1 error using the False Discovery Rate procedure.

### 3.3 Hypothesis 2 birth control and hormone therapy use

#### 3.3.1 Birth control

We hypothesized that timing and duration of birth control and menopausal hormone therapy would be associated with cognitive performance. More than three quarters of women in our sample reported a history of birth control use (*n* = 351, 76.6%). The average age of starting birth control was 21.7 (SD = 4.7), with an average duration of 6.4 years (SD = 7.0). These analyses were conducted in all women in the sample, regardless of oophorectomy status. Adjusting for age, education, SES, and *APOE4* carrier status, (Table 4) birth control use was significantly associated with better performance on the MoCA (r^2^ change = 0.008), and tests of working memory (r^2^ change = 0.01), and executive function/attentional control (r^2^ change = 0.010). In follow up models of those who reported birth control use, neither duration of birth control use (p range 0.244–0.848), nor age at time of starting birth control was associated with cognitive performance in any domain (p range 0.12–0.70), adjusting for age, education, SES, and *APOE4* carrier status.

**TABLE 4:** Association of birth control use with cognitive performance, adjusting for covariates.

	MOCA total		Episodic memory		Processing speed		Working memory		Executive function/ attentional control		Visuo-spatial processing	
	**β**	***P***	**β**	***P***	**β**	***P***	**β**	***P***	**β**	***P***	**β**	***P***
Age	-0.128	0.004	-0.135	0.002	-0.289	<0.001	-0.262	<0.001	-0.301	<0.001	-0.216	<0.001
Education	0.220	<0.001	0.248	<0.001	0.187	<0.001	0.262	<0.001	0.220	<0.001	0.310	<0.001
SES	0.166	<0.001	0.126	0.008	0.218	<0.001	0.204	<0.001	0.196	<0.001	0.174	<0.001
*APOE4* carrier	-0.130	0.003	-0.114	0.011	-0.045	0.298	-0.065	0.121	-0.039	0.353	-0.028	0.502
Birth control (yes = 1, no = 0)	0.093	0.037	0.086	0.054	0.083	0.052	0.102	0.016	0.103	0.015	0.078	0.066
Model adjusted R^2^	0.141	–	0.143	–	0.199	–	0.237	–	0.217	–	0.211	–

Corrected for type 1 error using the False Discovery Rate procedure.

#### 3.3.2 Menopausal hormone therapy

While 75.3% (*n* = 347) reported ever experiencing hot flashes or night sweats, only 39.5% (*n* = 182) reported a history of hormone therapy use. Of those reporting menopausal hormone therapy use, only 9.0% (*n* = 41) reported use in the last 3 months. Excluding individuals who underwent oophorectomy prior to natural menopause, the average age of reported natural menopause was 51.3 years (SD = 5.3). Among those who underwent natural menopause and reported hormone therapy use, the average starting age was 48.4 (SD = 6.9), with an average duration of 2.7 years (SD = 6.4). The average discrepancy between age at naturally occurring menopause and age at start of menopausal hormone therapy was 2.2 years prior to age at natural menopause (SD = 7.2).

Including only women who did not have oophorectomy prior to natural menopause, we estimated the effect of hormone therapy use adjusting for duration of use, age at starting hormone therapy, and distance between natural menopause and age at starting hormone therapy (Table 5). These models adjusted for age, education, SES, *APOE4* carrier status, and use of birth control medications. None of the variables related to hormone therapy duration or timing were significantly associated with processing speed, working memory, or executive function/attentional control. For the MoCA and episodic memory, associations of hormone therapy use or duration between hormone therapy start and menopause, were no longer significant after adjustment for type 1 error using the False Discovery Rate procedure (Benjamini and Hochberg, 1995). For visuospatial processing, duration of hormone therapy use was no longer associated with performance in this domain after correction for type 1 error using the False Discovery Rate procedure (Benjamini and Hochberg, 1995).

**TABLE 5:** Association of hormone therapy with cognitive performance, adjusting for covariates, excluding individuals undergoing oophorectomy prior to natural menopause.

	MoCA total		Episodic memory		Processing speed		Working memory		Executive function/attentional control		Visuo-spatial processing	
	**β**	***P***	**β**	***P***	**β**	***P***	**β**	***P***	**β**	***P***	**β**	***P***
Age	-0.157	0.058	-0.228	0.008^	-0.366	<0.001	-0.362	<0.001	-0.409	<0.001	-0.300	<0.001
Education	0.252	0.005	0.023	0.801	0.074	0.390	0.048	0.576	0.101	0.235	0.112	0.196
SES	0.100	0.248	0.158	0.076	0.257	0.003	0.219	0.010^	0.214	0.012^	0.191	0.026^
*APOE4* carrier	-0.191	0.024^	-0.209	0.016^	-0.015	0.853	-0.101	0.216	0.008	0.923	-0.093	0.258
Hormone therapy (yes = 1, no = 0)	0.186	0.028^	0.085	0.321	-0.041	0.619	0.040	0.622	-0.003	0.967	0.086	0.296
Duration of HT (years)	0.153	0.087	0.132	0.148	0.036	0.684	0.155	0.075	0.054	0.535	0.198	0.025^
Age started HT (years)	-0.022	0.856	-0.006	0.965	0.092	0.449	0.157	0.192	0.131	0.275	0.160	0.186
Menopause age minus HT start age	-0.258	0.038^	-0.275	0.031^	-0.065	0.595	-0.154	0.202	-0.083	0.486	-0.199	0.103
Birth control use (yes = 1, no = 0)	0.081	0.344	0.085	0.333	0.111	0.192	0.063	0.454	0.100	0.228	0.019	0.821
Adjusted R^2^	0.184	–	0.145	–	0.205	–	0.227	–	0.235	–	0.212	–

^No longer statistically significant after correction for type 1 error using the False Discovery Rate procedure. HT, hormone therapy.

### 3.4 Hypothesis 3 *APOE* interactions on cognitive performance

We hypothesized that *APOE4* carriers would differ in their associations between oophorectomy, hormone therapy, and cognitive performance. In our sample, 28.4% of women carried one or more *APOE4* alleles. Independent samples *t*-tests indicated that *APOE4* carriers differed from non-*APOE4* carriers on their mean performance on MoCA and the episodic memory domain [MoCA t (df) = 2.98 (453), *p* = 0.003, episodic memory t (df) = 2.45 (453), *p* = 0.015]. We did not observe mean differences in performance for any of the other cognitive domains (ps range from 0.142 to 0.419). The interaction between *APOE4* carrier status and oophorectomy did not reach the level of statistical significance for any of the cognitive outcomes, adjusting for covariates (MoCA β = −0.077, *p* = 0.202; episodic memory β = −0.067, *p* = 0.275; processing speed β = −0.077, *p* = 0.191; working memory β = −0.085, *p* = 0.140; executive function/attentional control β = −0.082, *p* = 0.158; visuospatial β = −0.080, *p* = 0.168).

The interaction between *APOE4* carrier status and birth control use was not significant for any of the cognitive outcomes adjusting for covariates (MoCA β = −0.167, *p* = 0.112; episodic memory β = −0.154, *p* = 0.144; processing speed β = 0.131, *p* = 0.196; working memory β = 0.003, *p* = 0.974; executive function/attentional control β = 0.123, *p* = 0.219; visuospatial β = −0.075, *p* = 0.457).

The interaction between *APOE4* carrier status and hormone therapy use was not significant for any of the cognitive outcomes adjusting for covariates (MoCA β = −0.099, *p* = 0.130; episodic memory β = −0.122, *p* = 0.062; processing speed β = −0.055, *p* = 0.385; working memory β = −0.056, *p* = 0.368; executive function/attentional control β = −0.031, *p* = 0.617; visuospatial β = −0.062, *p* = 0.320).

## 4 Discussion

Our study demonstrates that estrogen exposure in young adulthood, in the form of birth control, and hormone therapy in women undergoing oophorectomy before natural menopause are both associated with better cognitive function post-menopause. Further, the effects were demonstrated across multiple cognitive domains, not simply the verbal memory domain commonly assessed because of its role in detection of Alzheimer’s disease.

Our results suggest that oophorectomy prior to natural menopause was not associated with worse performance in any cognitive domain, when accounting for use of hormone therapy. Although the null finding contradicts our original hypothesis, the timing of surgery and hormone therapy relative to natural menopause onset provide a reasonable explanation that is consistent with the literature. That is, among women who underwent oophorectomy, use of hormone therapy was a significant predictor of better cognitive function in multiple domains, though effect sizes were small. In our sample, the majority of the women who underwent oophorectomy reported use of hormone therapy within 2 years before or after the surgery. Thus, any negative impact of oophorectomy on cognition may have been mitigated by hormone therapy use (Crawford, 2022; Phung et al., 2010; Rocca et al., 2014, 2021). The timing of oophorectomy relative to natural menopause has been recognized to be important for any association with cognitive outcomes. The recent meta-analysis by Andy et al. (2024) suggested that hormone therapy was most beneficial for women who had undergone surgically induced menopause, particularly at a young age. The average woman in our sample undergoing oophorectomy did so at an age relatively close to the natural age of menopause (46 vs. 51) and more than half were treated with hormone therapy at the same age. As such, our sample of individuals who underwent oophorectomy may have been at lower risk of poor cognitive effects that have been associated with this procedure compared to those who undergo oophorectomy at an earlier age, or without hormone therapy after the procedure. Because we were unable to distinguish between unilateral and bilateral oophorectomy, it is possible that some proportion of individuals in the sample underwent a unilateral oophorectomy and may therefore have continued to be exposed to estrogen produced by the remaining ovary.

Birth control medication use, with an average age of onset of 22, was associated with better performance on the MoCA, tests of working memory, and executive function/attentional control in older adulthood, though duration of use or age of onset of use were not significantly associated with performance. This is consistent with previous reports (Karim et al., 2016) associating use of hormonal contraceptives with cognitive benefits and in support of the hypothesis that greater young adult estrogen exposure has a positive effect on later life cognitive function (Matyi et al., 2019; Ryan et al., 2009). In contrast, after adjustment for covariates and correction for type 1 error, menopausal hormone therapy use was not associated with cognitive performance in our sample. This is consistent with a recent meta-analysis showing that, overall, hormone therapy is not associated with global cognitive performance (Andy et al., 2024). In contrast with prior literature, we did not find strong evidence that timing and duration of hormonal menopause therapy influenced the association with cognitive performance in our sample. We note that our ability to estimate the timing of hormone therapy use was complicated by lack of detailed participant recall of hormone formulations, dosage, routes of administration, and timing. Lord et al. (2009) warn that self-report measures of estrogen exposure are biased by poorer recall of events further in the past. Furthermore, participant reporting of natural menopause versus surgically induced menopause indicated a lack of clear understanding of the physiology of menopause (e.g., reporting both oophorectomy prior to the typical age of menopausal onset and natural menopause). Research should continue to address challenges in recall bias, perhaps by using more detailed medical record data. The Stages of Reproductive Aging Workshop (STRAW) (Harlow et al., 2012) developed guidelines for assessing reproductive staging that is recommended for standardization across studies. As a tertiary outcome in the IGNITE study, STRAW staging was not available for our sample. Anecdotal evidence from women’s health care providers suggests that current medical records documentation of menopause and its treatment is poor and agreement between physician and self-reported characterization of menopause is weak. Future studies could contribute to better understanding and utilizing medical records documentation for the purposes of studying and treating menopause symptoms.

Finally, we hypothesized that *APOE4* carrier status would interact with indicators of estrogen exposure on cognitive performance. In our sample, *APOE4* carriers consistently had worse performance on the dementia screener (MoCA) and the episodic memory domain scores across analyses, which is consistent with the body of evidence tying *APOE4* genotype to Alzheimer’s pathological processes and patterns of cognitive performance that are consistent with memory declines (El Haj et al., 2016). We did not find any significant interactions between *APOE4* carrier status and oophorectomy, use of birth control, or hormone therapy that influenced cognitive performance scores. One possible explanation for a lack of findings is that effect sizes for genotypes on cognitive performance are notably small (Chabris et al., 2015) and our analyses were powered for medium effect sizes. This is sometimes called “the fourth law of behavior genetics” which describes that complex human behaviors are associated with many genetic variants, each of which accounts for a very small percentage of variability in the behavior. Findings of prior studies on the relationship between hormone therapy and cognition as it relates to *APOE* genotype have been mixed (Saleh et al., 2023; Yaffe et al., 2000) and we were unable to further elucidate these conflicting findings. Some possible explanations for this include differences in study sample and study design. Specifically, the women in the Saleh et al. study differed from our study in that they were identified as being at increased risk for dementia. The Yaffe study was longitudinal, while our study was cross-sectional.

Our study has notable limitations. The association between birth control and cognitive performance might be confounded by other unmeasured variables, for example complications of pregnancy. Pre-eclampsia during pregnancy has been associated with poorer late life cognitive outcomes (Fields et al., 2017). As the primary aims of the study were unrelated to reproductive history, we were unable to measure pregnancy history, menopause history, or hormone therapy history in greater detail. Another key limitation is the cognitively unimpaired sample who were required to be in physical condition to exercise safely and have no contraindications to MRI to be enrolled in the study. Thus, we are unable to draw conclusions about the relationships under investigation among participants at greater risk for cognitive decline or dementia. The cross-sectional, retrospective self-report of the timing of hormone therapy limits our ability to accurately evaluate potential non-linear effects of age and timing of hormone therapy in its effects on subsequent cognitive performance, which could have obscured our ability to detect effects. Furthermore, the cross-sectional nature of the study in late adulthood limits our ability to draw conclusions about causality, mechanisms occurring in midlife, or long-term impacts of earlier life hormone exposure. Finally, we did not have adequate statistical power to compare heterozygous to homozygous *APOE4* carriers nor to add all possible confounding factors such as measures of diet or physical activity, though the role of these factors should be explored in future research.

Our study uniquely adds comprehensive and robust assessment of a wide array of cognitive domains rather than single tests or limited to the verbal memory domain, which is most typically assessed in prior research studies. Future directions might include creation of a risk score to combine various indicators of lifetime estrogen exposure, such as presence, duration, dosage, since each alone accounts for only a small amount of variance in the overall contribution to late life cognition, a very distal outcome. Additional investigation of contributions of lifetime estrogen exposure to change in cognition across the lifespan would add to our understanding of female-specific developmental implications related to stages including puberty, pregnancy, reproductive surgery, and menopause. It would be particularly beneficial to conduct this research in a more representative samples of women from varying racial and ethnic, sociocultural, geographic and economic backgrounds to ensure generalizability of findings. Imaging studies can help to link brain structure and function to our findings with cognitive assessment. For example, estrogen modulation of hippocampal function is likely play a role in memory consolidation (Frick et al., 2010). Innovations in the accurate assessment of reproductive and menstrual history would also greatly strengthen our ability to understand these lifelong associations. Finally, further exploration of causal mechanisms by which lifetime estrogen exposure may influence cognitive and brain function are needed and may include mitochondrial function, sarcopenia, and gut-brain axis (Jiang et al., 2019; Zhang et al., 2024).

## Data Availability

The original contributions presented in the study are publicly available. This data can be found here: DOI 10.17605/OSF.IO/AEZJ8.

## Appendix

**Appendix 1:** Sources of potential error in categorization of menopause status.

Final category	Explanation
Oophorectomy	We observed multiple patterns of reporting that included oophorectomy. Unilateral oophorectomy would not necessarily result in menopause. We did not collect data about unilateral vs. bilateral oophorectomy. Oophorectomy without hysterectomy, reported uncertain about natural menopause (*n* = 1). Classified as oophorectomy. Oophorectomy without hysterectomy, reported no natural menopause (*n* = 46). Classified as oophorectomy. Oophorectomy without hysterectomy, reported yes to natural menopause (*n* = 23). Classified as oophorectomy. Oophorectomy with hysterectomy, reported uncertain about natural menopause (*n* = 5). Classified as oophorectomy. Oophorectomy with hysterectomy, report yes to natural menopause (*n* = 30). Classified as oophorectomy.
Unclear	With an age range of 65–80, it is unlikely, but not impossible that women have not undergone menopause. Four women in the sample reported not having experienced natural or surgical cessation of menses. Two endorsed menopausal symptoms, two did not. Explanations for unclear menopause status could include: a) peri-menopausal status, b) lack of awareness of signs of menopause, c) health conditions that prevent observation of normal periods, including fibroids that cause bleeding, d) Endometrial ablation which reduces or leads to cessation of bleeding, e) use of birth control that results in cessation of bleeding, f) late menopause, three participants classified as unclear reported no menopausal symptoms, four reported menopausal symptoms, one reported current symptoms. Ages ranged from 65 to 77.
